# LPS Nephropathy in Mice Is Ameliorated by IL-2 Independently of Regulatory T Cells Activity

**DOI:** 10.1371/journal.pone.0111285

**Published:** 2014-10-24

**Authors:** Roberta Bertelli, Armando Di Donato, Michela Cioni, Fabio Grassi, Masami Ikehata, Alice Bonanni, Maria Pia Rastaldi, Gian Marco Ghiggeri

**Affiliations:** 1 Division of Nephrology, Dialysis, Transplantation and Laboratory on Physiopathology of Uremia, Giannina Gaslini Children Hospital, Genoa, Italy; 2 Institute for Research in Biomedicine, Bellinzona, Switzerland; 3 Department of Medical Biotechnologies and Translational Medicine, University of Milan, Milan, Italy; 4 Renal Research Laboratory, Fondazione Istituto di Ricerca e Cura a carattere Scientifico (IRCCS ) Ca’ Granda Ospedale Maggiore Policlinico and Fondazione D’Amico per la Ricerca sulle Malattie Renali, Milano, Italy; National Institutes of Health, United States of America

## Abstract

Immunosuppressive regulatory T cells (Tregs) have been hypothesized to exert a protective role in animal models of spontaneous (Buffalo/Mna) and/or drug induced (Adriamycin) nephrotic syndrome. In this study, we thought to define whether Tregs can modify the outcome of LPS nephropathy utilizing IL-2 as inducer of tissue and circulating Tregs. LPS (12 mg/Kg) was given as single shot in C57BL/6, *p2rx7^−/−^* and Foxp3^EGFP^; free IL-2 (18.000 U) or, in alternative, IL-2 coupled with JES6-1 mAb (IL-2/anti-IL-2) were injected before LPS. Peripheral and tissue Tregs/total CD4+ cell ratio, urinary parameters and renal histology were evaluated for 15 days. IL-2 administration to wild type mice had no effect on peripheral Tregs number, whereas a significant increase was induced by the IL-2/anti-IL-2 immunocomplex after 5 days. Spleen and lymph nodes Tregs were comparably increased. In *p2rx7^−/−^* mice, IL-2/anti-IL-2 treatment resulted in increase of peripheral Tregs but did not modify the spleen and lymph nodes quota. LPS induced comparable and transient proteinuria in both wild type and *p2rx7^−/−^* mice. Proteinuria was inhibited by co-infusion of human IL-2, with reduction at each phase of the disease (24 −48 and 72 hours) whereas IL-2/anti-IL-2 produced weaker effects. In all mice (wild type and *p2rx7^−/−^*) and irrespective of treatment (IL-2, IL-2/anti-IL-2), LPS was associated with progressive signs of renal pathologic involvement resulting in glomerulosclerosis. In conclusion, IL-2 plays a transient protective effect on proteinuria induced by LPS independent of circulating or tissue Tregs but does not modify the outcome of renal degenerative renal lesions.

## Introduction

Glomerulonephritis potentially derive from a direct immunomodulatory effect of circulating blood cells, antibodies, cytokines and other mediators on the kidney. [1], [2], [3] Autoimmune diseases are an example but there are also less defined conditions such as nephrotic syndrome, for which evidence for an immunomediated mechanism is accumulating in animal models and in humans. In fact, oxidants are known to mediate inflammation in experimental models [4], [5] and there is indirect evidence for oxidant hyperactivity in humans with nephrotic syndrome; [6], [7], [8], [9] co-stimulatory molecules may also be activated in some instance [10], [11]. The Lipopolysaccharide (LPS) model of proteinuria is of particular interest in studying the immunomodulatory link: mice lacking the B7-1 (a co-stimulatory molecule) are, in fact, protected from developing proteinuria [10] and proteinuria protection has been proposed, albeit with controversy [12], in nephrotic patients using the inhibitor of B7-1 molecule abatacept. [11]. There is convincing evidence that LPS is directly active on podocytes and is independent of T or B cells. Experiments in SCID mice, which are devoted of both cell lineages but still develop proteinuria after LPS, are central to this demonstration. [10].

Circulating cells deputed to regulation of the immune response, in particular CD4+, potentially play a regulatory role in LPS (and in other experimental and human nephropathies as well) if their level is increased by drugs. One possibility is that CD4+ exacerbate the glomerular damage by differentiating into Th17 or by recruiting macrophages and neutrophils [13] [14]; they may also act as negative feedback effectors of the adaptive immune response by differentiating into CD4+ CD25+ regulatory T cells (Tregs). [15] The thin demarcation between pro-inflammatory and anti-inflammatory pathways is crucial in determining the outcome in many renal diseases.

The enhancement of Tregs function represent an attractive therapeutic approach in treating many autoimmune and inflammatory renal disorders [16]. Positive results were obtained with Tregs expansion in experimental crescentic nephropathy [17] and in different models of nephrosis arising spontaneously [18] in Buffalo/Mna rats or induced by Adriamycin [19]. In both cases, Tregs have been directly infused in rats with experimental nephrosis [18], [19], or have been up-regulated with exogenous IL-2 [17]. A partial improvement of renal function and a reduction of tissue lesions were obtained.

Studies with IL-2 in humans are limited to cryoglobulinemia in which case infusion of low dose IL-2 was followed by an increase in Treg and was associated with clinical improvement [20].

In this study, we examined the effects of Tregs induction by IL-2 on the onset and progression of LPS nephropathy, a transient model of proteinuria [10]that determines renal lesions similar to focal segmental glomerulosclerosis in humans. Two models for IL-2 treatment were utilized on the basis of different cell subset activity of IL-2 when infused as free cytokine or otherwise coupled with anti-IL-2 JES6-1 antibodies (IL-2/anti-IL-2); in the latter case the IL-2/anti-IL-2 complex plays a selective effect on the IL-2-Rα chain (CD25) that is highly expressed in Tregs [21]. The role of ATP as potential inducer of the renal damage and/or as modulator of circulating and tissue Tregs was in parallel evaluated by utilizing mice lacking the P2×7 receptor and for this reason less prone to inflammatory stimuli [22].

## Materials and Methods

### Ethical statement

Experiments in mice were done according to the principles expressed in the Declaration of Helsinki and were approved by the Institutional Review Board of IRCCS S. Martino (Genoa) and by the local authorities according to the legal requirements.

### Animals

Six- to eight-week-old male C57BL/6 mice (weight 20–25 g) were purchased from Harlan Laboratories (Indianapolis, IN, USA). *P2rx7^−/−^* (B6.129P2-*P2rx7^tm1Gab^*/J) and “Foxp3^EGFP^” (B6.Cg-*Foxp3^tm2Tch^*/J) were from Jackson Laboratories (Bar Harbour, ME, USA) and were backcrossed to obtain *p2rx7^−/−/^*Foxp3^EGFP^ mice.

### IL-2 and IL-2/anti-IL-2 infusion

Where indicated, for 14 days before LPS treatment, mice were given daily an intra-peritoneal injection of human recombinant IL-2 (18.000 U in 100 µl) (Proleukin, Novartis, Basel, Switzerland). In the case of IL-2/anti-IL-2 complex, mice were treated for 5 days prior to LPS, with 1 µg mouse recombinant IL-2 previously mixed and incubated at 37°C for 30 minutes with 5 µg anti-mouse IL-2 monoclonal antibody (clone JES6-1) (Sigma Aldrich, St. Louis, MO, USA) as described by other Authors [21], [23].

### Experimental LPS nephropathy

For induction of LPS nephropathy, all mice were injected intra-peritoneal with 12 mg/Kg LPS (serotype 0111:B4, Sigma Aldrich, St Louis, MO, USA) dissolved in sterile LPS-free PBS in a total volume of 100 µl/mouse, or with an equal volume of sterile LPS-free PBS, as previously described by Reiser [10]; after 24, 48 and 72 hours urines were collected for monitoring proteinuria. Blood samples for determination of circulating Tregs were obtained at different times from IL-2 and/or IL-2/anti-IL 2 infusions. In the former case, blood samples were obtained at T0, T14 (corresponding to the intervals utilized for IL-2 induction of Tregs) and after 3 further days from LPS. In the case of IL-2/anti IL-2, blood was obtained at T0, T5 days (corresponding to the interval utilized for Treg induction) and after 3 further days from LPS. Spleen and lymph nodes Tregs were determined at the end of the experiments corresponding to +T17 from IL-2 infusion and +T8 from IL-2/anti-IL-2.

For renal pathology, mice were sacrificed 7 days from LPS.

### Determination of albumin

Urinary albumin was separated by 10% SDS-PAGE, and identified by immunoblotting, using a goat polyclonal anti-mouse albumin antibody (Santa Cruz Biotechnology Inc, Dallas, TX, USA) and an alkaline phosphatase-conjugated anti-goat as secondary antibody (Santa Cruz Biotechnology Inc, Dallas, TX, USA). The blot was then developed with nitro blue tetrazolium chloride/5-bromo4-chloro-3-indolyl phosphate (NBT/BCIP) reagents (Roche Diagnostics GmbH, Mannheim, Germany). In order to quantify the mouse albumin bands the blots were digitalized and a densitometry was performed by using the software NIH ImageJ v. 6.4 (freeware, NIH, Bethesda, MD, USA). A known quantity (1–3 µg) of mouse albumin (Sigma-Aldrich, St. Louis, MO, USA) was used as reference.

### TLR4 western blot

Where indicated we used an anti-TLR4 monoclonal antibody (sc-293072 Santa Cruz Biotechnologies, Dallas, TX, USA) and a goat anti-mouse alkaline-phosphatase conjugated antibody as secondary antibody (sc-2008 Santa Cruz Biotechnolgies, Dallas, TX, USA).

### Flow cytometry for Tregs analysis

For determination of Treg number, peripheral blood was collected at different times by retro-orbital bleeding; PBMC were isolated using Erythrocyte Lysing Reagent (Uti-Lyse, Dako, Glostrup, Denmark), according to manifacturer’s instructions, stained with anti- mouse CD4-PE monoclonal antibody (Santa Cruz Biotechnology, Dallas, TX, USA) for 20 minutes at room temperature and analyzed by flow cytometry technique on BD FACSCanto II instrument with FACSDiva Software. Spleen and draining lymph nodes specimens were harvested at 72 hours after LPS administration, minced and pressed onto 40 µm Cell Strainers (BD Falcon, San Josè, CA, USA), to obtain a monocellular suspension; for identification of double–positive CD4+ Foxp3+ cells, after erythrocyte lysis with ACK buffer, cells were stained with PE-coniugated anti-mouse CD4 (Santa Cruz Biotechnology Inc, Dallas, TX, USA). and analyzed by flow cytometry as above described.

### Histochemistry

Renal tissues for light microscopy were fixed in 4% buffered paraformaldehyde and embedded in paraffin; 3 µm sections were cut, deparaffinised, rehydrated, and stained with Hematoxilin/Eosin, PAS, and Trichrome according to standard techniques.

Evaluation of main histological parameters (i.e. mesangial hypercellularity, mesangial matrix expansion, segmental glomerulosclerosis, tubular casts, tubular atrophy, interstitial infiltration, interstitial fibrosis) was performed semiquantitatively by two independent observers not aware of the mouse genotype and treatment. Glomerular evaluation was performed on a minimum of 40 glomeruli per section.

## Results

### LPS induced nephropathy

**Figure 1:**
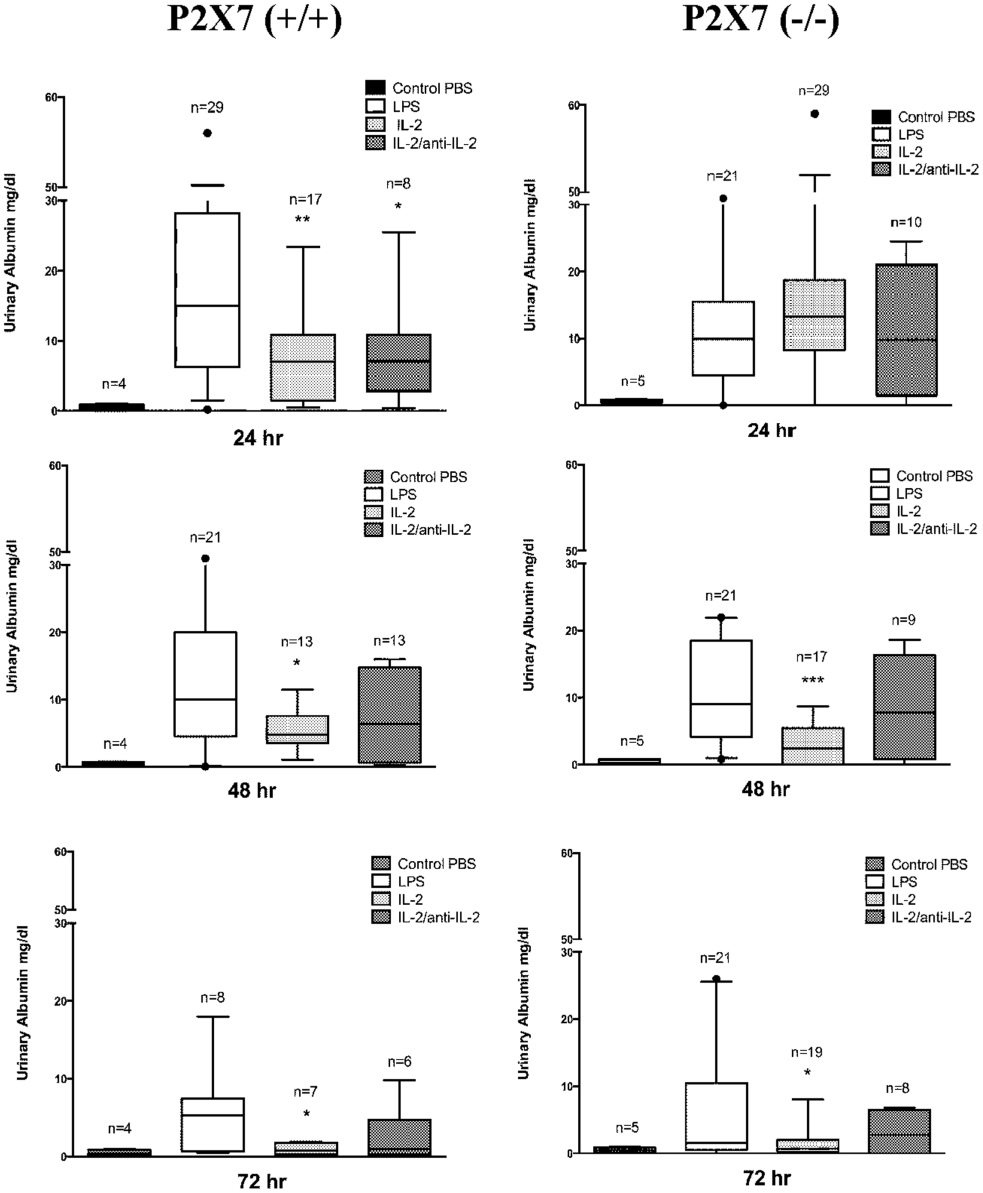
Proteinuria outcome in LPS mice. Urinary albumin levels were evaluated with immune-western after LPS (12 mg/Kg) treatment (24 hrs, 48 hrs and 72 hrs after treatment) in wild C57BL/6 and in P2×7^−/−^ mice. In some cases and in both experimental groups (WT and P2×7^−/−^) IL-2 (18.000 U) and/or IL-2/anti-IL-2 were infused prior LPS to regulate circulating and tissue Tregs (see Figure 3). * = p≤0.05, ** = p≤0.005, *** = p≤0.0005.

**Figure 2:**
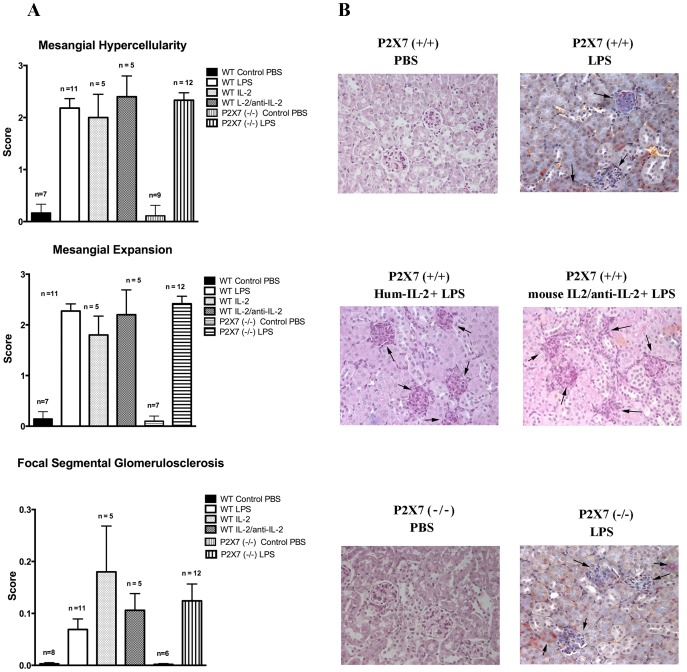
Histology features. Classical histology (Ematoxylin Eosin, PAS) of kidney biopsies was evaluated after several times from LPS infusion (24hrs, 72 hrs and 7 days). Three main features were observed: 1-mesangial hypercellularity, 2-mesangial expansion and 3-focal segmental glomerulosclerosis that were determined in a semi-quantitative basis (score 0–3 for the former two parameters, score 0–0.3 for glomerulosclerosis). Relevant results observed after 7 days are presented in this figure (A). Specific patterns are shown in (B); stains are hematoxylin eosin for all with the exception of Masson Blue stain for LPS alone.

Intraperitoneal infusion of LPS in mice triggers an inflammatory cascade and is sometimes associated with lethal septic shock. LPS also induces variable and transient proteinuria, typically beginning 24 hours after the infusion and lasting for 72 hours when a rapid decline is followed by normalization. In our hands albuminuria varied from 15 mg/dl at 24 hours to 10 mg/dl at 48 hours and 5,3 mg/dl at 72 hours (Figure 1). Renal histology was characterized by mesangial proliferation and mesangial matrix espansion that were evident at 24 hrs from LPS infusion and increased over time. After 7 days also segmental glomerulosclerosis was present and relevant in terms of glomeruli involved (30–50%)(Figure 2).

### LPS induced nephropathy is not dependent on extracellular ATP

The ATP gated ionotropic P2×7 receptor participates in NLRP3 inflammasome activation and in sensing extracellular ATP released by tissue damage. Its blockade was shown to attenuate murine lupus nephritis [24]. In addition it was recently shown that Tregs lacking P2×7 are more stable and efficient as immunosuppressive cells in inflammatory bowel disease [25]. To address whether ATP release and signalling via P2×7 receptor were involved in LPS induced nephropathy, we injected LPS in *p2rx7^−/−^* mice. However, lack of P2×7 did not result in amelioration of proteinuria after LPS treatment (Figure 1) and the outcome of proteinuria maintained the same characteristics of LPS treated wild type mice with spontaneous remission occurring after 72 hours from LPS infusion. Renal histology was not modified as well (Figure 2).

### Proteinuria is modified by human IL-2 in LPS nephropathy

LPS nephropathy was inhibited by co-infusion of human IL-2, in which case proteinuria was reduced at each phase of the disease (7 mg/dl at 24 hours after LPS, 4,8 mg/dl at 48 hours and 0,8 mg/dl after 72 hours) (Figure 1). Co-infusion of mouse IL-2 coupled with JES6-1 mAb (IL-2/anti-IL-2) produced weaker but still significant reduction of proteinuria at each phases of the disease (Figure1).

In *p2rx7^−/−^* mice, the effect of IL-2 pre-treatment on proteinuria was negligible at 24 hours (13,3 mg/dl) while being evident after 48 and 72 hours from LPS, (e.g. 2,4 mg/dl and 0,7 mg/dl respectively (Figure 1); thus, in absence of the P2×7 receptor, IL-2 seems to influence disease progression without modifying proteinuria at the onset.

Once again, this protective effect was less evident in mice pre-treated with IL-2/anti-IL-2 (Figure 1): in this case, persistent and comparable levels of proteinuria were found in *p2rx7^−/−^* mice, irrespectively of cytokine pre-treatment, in the entire observation period.

Histological parameters were similar in all phases of proteinuria and were not modified by IL-2 nor by IL-2/anti-IL-2 infusion (Figure 2A, B).

### Treg levels are regulated by IL-2/Anti-IL-2

**Figure 3:**
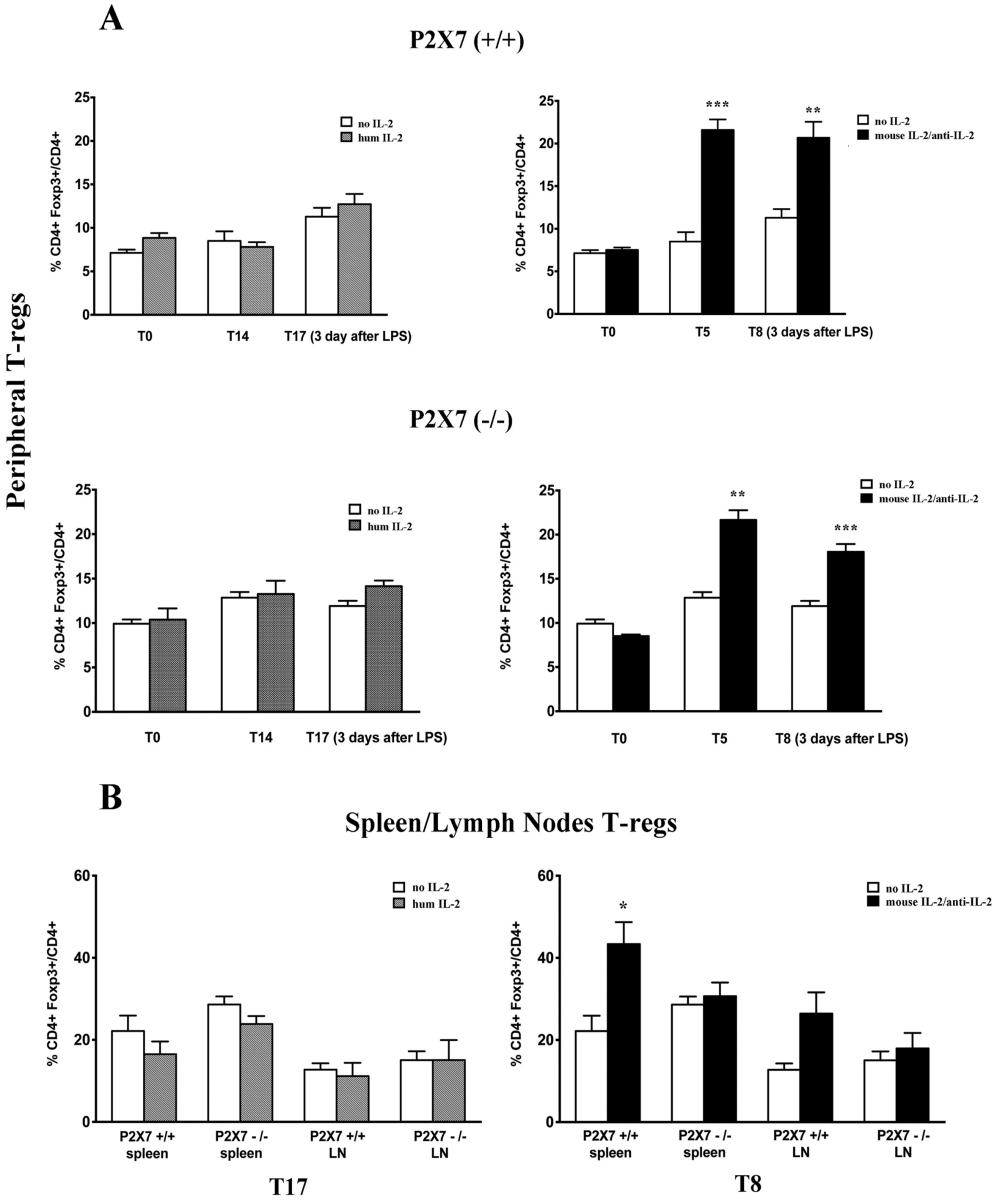
Treg level regulation by IL-2 and IL-2/anti-IL-2. Peripheral (A) and spleen/lymph nodes (B) Tregs levels were evaluated at several intervals after IL-2 and IL-2/anti-IL-2 treatment and after LPS in both WT and P2×7^−/−^ mice. In the case of IL-2, determination of circulating and tissue Tregs was done after 7 and 14 days from IL-2 that is the time potentially required to achieve a regulatory effect of the cytokine [21].; Tregs were also determined 3 days after LPS that means 17 days from IL-2 treatment. The time required for a regulatory effect of IL-2/anti-IL-2 is instead 5 days. Treg levels were accordingly determined at this time (i.e. 5 days after IL-2/anti-IL-2) and after 3 days from LPS corresponding to 8 days of treatment. * = p≤0.05, ** = p≤0.005, *** = p≤0.0005.

For the experiments on Tregs regulation by IL-2, we utilized Foxp3^EGFP^ mice that allow easy detection of Tregs in circulation and tissues due to spontaneous fluorescence. As shown in Figure 3A, IL-2 administration to wild type mice had no effect on peripheral Tregs number, whereas a significant increase in CD4+ Foxp3+/total CD4+ cell ratio was induced in peripheral blood by the IL-2/anti-IL-2 (i.e 21,6±1.23 compared to 9.04±0.28 in untreated animals) after 5 days from infusion. The same Tregs number remained elevated and was not modified by LPS (Figure 3A). Analysis of tissue Tregs at 72 hours after LPS treatment confirmed the increment of Tregs produced by IL-2/anti-IL-2 in spleen and lymph nodes (Foxp3+/CD4+ ratio 43.35±5.34 vs. 22.15±3.77) (Figure 3B). Once again, IL-2 did not significantly modify this value at any time.

The effect of IL-2/anti-IL-2 on peripheral Tregs was evident also in *p2rx7^−/−/^*Foxp3^EGFP^ mice (21.7±1.1 vs.10.6±0.4 in untreated mice) with a slight reduction after LPS treatment (18.06±0.87 in LPS treated versus 21.66±1.10 in mice before treatment) (Figure 3A). In spleen and in lymph nodes of *p2rx7^−/−/^*Foxp3^EGFP^ mice the positive regulation of IL-2/anti-IL-2 was absent (Figure 3B).

### Urine TLR-4 levels are modified by IL-2

**Figure 4:**
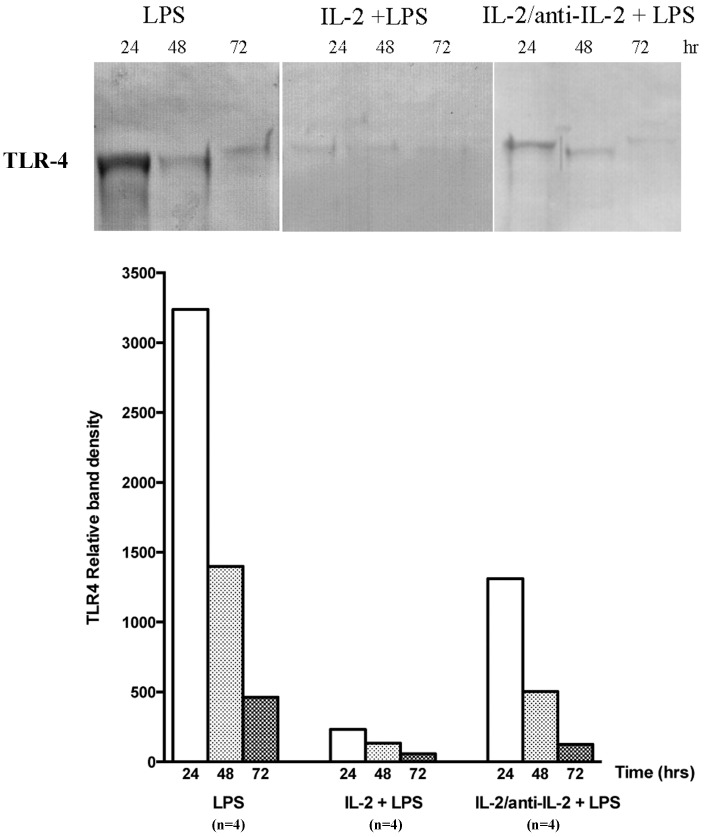
TLR-4 urine levels. Urine deriving from the same mice of the above experiments were utilized for determining levels of TLR-4, a molecule that interacts with LPS [10]. After LPS infusion, urine TLR-4 increased dramatically and then decreased in the following 72 hours. In mice treated with the combination of IL2 and LPS, urine TLR-4 levels were reduced by 1200% compared to LPS alone and then normalized in the 72 hours after. IL-2/anti-IL-2 produced intermediate effects.

LPS infusion in mice markedly increased urinary levels of TLR-4 during the 24 hours after infusion after then decreased by 80% after 72 hours (Figure 4). Combination of IL-2 and LPS produced a near normalization of urinary TLR-4 levels that after 24 hours from LPS were only slightly increased and after 72 hours only minimally detectable. IL-2/anti-IL-2 produced intermediate effects: after 24 hours from LPS, urinary TLR-4 levels were 40% than in mice treated with LPS alone and then decreased following the same outcome.

## Discussion

This work was aimed at addressing a possible protective role of Tregs in experimental nephropathy induced by LPS that is a model of transient proteinuria associated with foot process effacement, resembling human minimal change nephrosis [10], [26], [27] and evolving to glomerulosclerosis. LPS is sensed in podocytes by Toll-like receptor 4 (TLR-4) and up-regulates the co-stimulatory molecule B7-1 [28], [29]. From one side, the consequence is activation of an immune response, from the other is the reorganization of the actin cytoskeleton producing effacement of podocyte slit-diaphragm and proteinuria [10]. Overall, LPS nephropathy represents a suitable model to study the link between the innate immune response (TLR-4/B7-1) and the kidney and is, more in general, considered a reliable approach to study mechanisms of nephrotic syndrome. Studies addressing the direct effect of LPS on B7-1 are of particular interest since B7-1 activation in podocyte may be blunted by using the inhibitor of B7-1 molecule abatacept. [11], [12] Activation of B7-1 has also been shown in human beings with nephrotic syndrome that potentially suggests abatacept could be used in these patients [27], [30].

Few studies focused on the long-term effect of cytoskeleton re-organization in podocytes as modified by LPS. We know from familial cases of genetic nephrotic syndrome that mutations in cytoskeleton components lead to degeneration of glomerular tuft even if proteinuria is often mild. [31], [32], [33], [34] We could show here that, in spite of transient proteinuria, renal histology after LPS evolves in a few days to extensive glomerulosclerosis (30–50% of glomeruli). This is a new finding that strengthens LPS as a model of renal glomerulosclerosis (FSGS) and mimics what happens during years in patients carrying genetic variants of cytoskeleton genes [35].

A potential protective role of Tregs on LPS nephropathy was suggested by results in other experimental nephrosis (i.e in Buffalo/Mna rats and in Adriamycin nephrosis) that are, in the same way of LPS, two recognized models of proteinuria leading to glomerulosclerosis and renal failure [18] [19]. For enhancing Tregs function we utilized IL-2 since this cytokine plays an important role in Tregs growth, survival and activity in both mice and humans. IL-2 was utilized as free cytokine or in a complex with the JES6-1 anti-IL-2 antibody (i.e. IL-2/anti-IL-2), which plays different effects on T cell lineages. In fact, IL-2 activates CD4+, CD8+ T cells, Tregs and NK cells through different regions of the same IL-2 receptor composed of three subunits, i.e. α, β and γ that are differently expressed in CD8+ and NK cells versus Tregs [36]. Free IL-2 activates both the high affinity and the intermediate affinity receptor IL-2Rβ chain that is specific for memory CD8+ and NK cells, whereas when coupled with JES6-1 the IL-2 binding is restricted to the IL-2Rα chain (CD25) that is highly expressed on Tregs [21]. The effect of IL-2/anti-IL-2 complex on CD25- (hence on CD8+ and NK cells) is null, whereas it is maximal on Tregs. Indeed, Tregs expansion by IL-2/anti-IL-2 is protective in experimental models of allergy [16] and for the kidney it has been utilized with positive results in experimental crescentic glomerulonephritis [17] and in the renal ischemia-reperfusion injury [37]. As second model of protection from immunopathological damage, we exploited mice lacking the P2×7 receptor in which responsiveness of innate immune cells to tissue damage is weakened and Tregs are more stable and less prone to convert into pro-inflammatory cells [25], [38]. Moreover, podocytes silenced for P2×7 present a blunt response of inflammasome proteins to LPS suggesting some protection. [39].

As expected, Tregs were increased both in peripheral blood and secondary lymphoid organs (spleen and lymph nodes) after infusion of the IL-2/anti-IL-2 complex while a minimal increase was observed in mice receiving IL-2 alone [21]. The effects of both compounds (i.e. IL-2 and IL-2/anti-IL-2) on proteinuria were, instead, similar with IL-2 playing the most substantial protective effect. In contrast, lack of P2×7 activity did not influence proteinuria implying that ATP is not directly involved in the podocyte damage. This requires further study since it is in apparent contrast with what already reported on a blunted podocyte damage by LPS is when P2×7 is deleted. [39].

We observed a clear dichotomy between Tregs (highest numbers in mice treated with IL-2/anti-IL-2) and proteinuria (minimal in mice treated with IL-2). Altogether these results suggest that the protective effect of IL-2 is not linked to enhancement of Tregs functional activity. On the other hand, looking at renal histology it is clear that all parameters evaluated (i.e. mesangial hypercellularity and matrix expansion) were not modified by IL-2 and the same amount of glomerulosclerosis after 7 days from LPS was notable in all mice in spite of different treatments. Once again, there is a dichotomy between proteinuria and major renal modifications that implies IL-2 effect on proteinuria is ancillary to the mechanism of glomerulosclerosis and of limited importance. One possibility is that IL-2 modifies other factors directly linked to proteinuria without affecting the overall process of sclerosis, glomerular hemodynamics representing a potential candidate [40]. Modification of other immunocompetent cells and/or molecules should be considered. NK and CD4+/CD8+ cells are, in fact, induced by IL-2 and potentially modify B7-1 expression in podocytes by secreting soluble mediators (IL-10, TGF β and CTLA-4). Actually, we observed a marked decrease of TLR-4 urine excretion in mice treated with the combination of LPS and IL-2 that was instead, as known, very high in mice treated with LPS alone. In APC, LPS is a strong inducer of B7-1 through TLR-4 signaling [28] therefore, reduced TLR-4 would explain reduced B7-1 activation and LPS toxicity. While, this could explain the acute effect on proteinuria of IL-2 several and crucial aspects related to glomerulosclerosis remain unexplained. Historical data in human beings and in experimental nephrosis [41], [42] suggest that renal evolution to glomerulosclerosis and interstitial fibrosis (that are the two hallmarks of progressive renal lesions) is independent to any acute damage and is unique for several renal pathologies (from toxic, to immununologic, metabolic, etc.) mostly linked to hemodynamic conditions. It is possible that similar mechanisms are involved in accelerating progression to sclerosis in mice with LPS nephropathy.

Therefore, the results of our study do not support the general concept of Tregs mediated protection in animal models of immune-mediated renal diseases. Induction of Tregs attenuated proteinuria in Buffalo/Mna rats, a model of spontaneous nephrotic syndrome associated with focal segmental glomerulosclerosis [18] and also led to regression of these lesions in post-transplant recurrence of the disease. In a similar way, transfer of Foxp3-transduced T cells reduced proteinuria and renal glomerulosclerosis in rats treated with Adriamycin, a murine model of chronic proteinuria leading to renal failure [19]. Finally, administration of the same IL-2/anti-IL-2 antibodies used in our study was renoprotective in mice treated with Adriamycin showing less histological injury, better renal function and less inflammation [43].

In spite of the inconsistencies mentioned above, the findings on IL-2 protection in LPS nephropathy come in the mid of a road where low-dose IL-2 is considered for human use [44]. The most remarkable application is in patients with HCV related vasculitis in which case resolution of infection correlates with recovery of Tregs levels [20]. Circulating levels of IL-2 and Tregs in nephrotic syndrome have been also investigated in the past in view of the general concept that nephrotic syndrome is a T cell disorder [45], [46]. Studies on Tregs and more in general on cytokines in humans are scanty. Araya and col [47] described an impaired Tregs function in patients with steroid resistant nephritic syndrome but the analysis of IL-2 levels were inconsistent for a role of this cytokine.

In conclusion, the results of this study confirm the positive regulation of Tregs by IL-2/anti-IL-2 complex while IL-2 alone had no effect. They also show that proteinuria in LPS nephropathy is not sensitive to modulation by Tregs while it can be reduced by infusion of IL-2. Renal lesions of glomerulosclerosis were not modified by IL-2 and IL-2/anti-IL-2 implying that other factors influence renal homeostasis following LPS. Finally, mice lacking P2×7 had the same proteinuria and renal lesions of wild-type mice treated with LPS suggesting that both extracellular ATP via P2×7 stimulation and Tregs stability by P2×7 blockade are not crucially involved in proteinuria/renal lesions in this model.
